# Distinct Ca^2+^ pools regulate NADPH oxidase 2 activation driving Ca^2+^-independent mitochondrial ROS formation and mitochondrial permeability transition in arsenic trioxide-treated NB4 cells

**DOI:** 10.1007/s00204-026-04328-9

**Published:** 2026-04-13

**Authors:** Andrea Guidarelli, Andrea Spina, Gloria Buffi, Mara Fiorani, Orazio Cantoni

**Affiliations:** Department of Biomolecular Sciences, University of Urbino Carlo Bo, Urbino, Italy

**Keywords:** Arsenic trioxide, Acute promyelocytic leukemia, Inositol 1,4,5-trisphosphate receptor, Ryanodine receptor, NADPH oxidase 2, Mitochondrial superoxide, Mitochondrial permeability transition, Apoptosis

## Abstract

Clinically relevant concentrations of arsenic trioxide (ATO) induce apoptosis in NB4 cells through a complex, yet poorly defined interplay between endoplasmic reticulum—derived Ca^2+^ signalling and mitochondrial oxidative stress. This study enhances our understanding of these mechanisms by demonstrating that exposure to 1 µM ATO initiates a biphasic Ca^2+^ release: an initial flux from inositol 1,4,5-trisphosphate receptors (IP₃Rs), followed by a secondary release via ryanodine receptors (RyRs). Unlike IP_3_R-derived Ca^2+^, the fraction of the cation released through RyRs is subsequently taken up by mitochondria. Notably, IP_3_R-derived Ca^2+^ uniquely activates NADPH oxidase 2 (NOX 2), a key event leading to the downstream generation of mitochondrial superoxide (mitoO_2_^.−^). Importantly, mitochondrial Ca^2+^ accumulation itself is not required for mitoO_2_^.−^ emission. ATO-induced genomic DNA strand breaks are mediated by NOX 2-derived reactive oxygen species (ROS), both directly and indirectly, through the subsequent induction of mitochondrial ROS formation. Furthermore, mitochondrial uptake of RyR-derived Ca^2+^ is essential for triggering the mitochondrial permeability transition and the ensuing apoptotic cell death. Although sodium arsenite elicited comparable effects on Ca^2+^ homeostasis, it promoted mitoO_2_^.−^ generation via a distinct, NOX 2-independent pathway that relied on RyR-mediated mitochondrial Ca^2+^ accumulation. Thus, in NB4 cells, ATO exposure orchestrates a functional crosstalk between discrete Ca^2+^ sources to regulate a cascade of events culminating in NOX 2 activation, mitoO_2_^.−^ production, and initiation of the mitochondrial apoptotic pathway.

**Supplementary Information:**

The online version contains supplementary material available at 10.1007/s00204-026-04328-9.

## Introduction

Acute promyelocytic leukemia (APL), characterized by the expression of the promyelocytic leukemia-retinoic acid receptor-α (PML-RARα) fusion oncoprotein (de The et al. 1990; Melnick and Licht 1999), exhibits unique sensitivity to arsenic trioxide (ATO), a property linked to the ability of the arsenic compound to promote degradation of the fusion protein (Lallemand-Breitenbach et al. 2012; Shen et al. 1997; Zhu et al. 1997).

In these cells, clinically relevant concentrations of ATO (1–2 µM, (Westervelt et al. 2001; Zhang et al. 2010) cause the formation of reactive oxygen species (ROS) through NADPH oxidase 2 (NOX 2) activation, critically connected with the ensuing toxicity (Chou et al. 2004; Li et al. 2008; Wang et al. 2008), and this event is at least in part associated with PML-RARα degradation-induced NOX 2 inhibitory signaling (Li et al. 2008).

The same clinically relevant concentrations of ATO also induce mitochondrial superoxide (mitoO_2_^.−^) emission in APL cells (Jing et al. 1999; Korper et al. 2004; Kumar et al. 2014), leading to downstream mitochondrial permeability transition (MPT) and to the triggering of the mitochondrial pathway of apoptosis (Jing et al. 1999; Korper et al. 2004; Kumar et al. 2014).

Several lines of evidence suggest the involvement of PML-RARα fusion protein degradation in events upstream to mitoO_2_^−^. formation. For example, it has been proposed that ATO promotes disulfide bond formation and multimerization of PML-RARα, thereby causing mitochondrial instability associated with mitoO_2_^−^. emission (Jeanne et al. 2010). Mitochondrial aggregation has also been proposed as a mechanism associated with mitoO_2_^−^. formation (Hao et al. 2022).

We recently showed that, in NB4 cells, a widely employed APL cell line (Lanotte et al. 1991), NOX 2 activation is causally connected with downstream mitoO_2_^−^. formation, which indirectly also implies the involvement of PML-RARα.

Thus, ATO induced mitoO_2_^.−^ emission was suppressed via pharmacological inhibition of NOX 2, or siRNA mediated downregulation of p47^phox^ (Spina et al. 2025), a critical subunit of the NOX 2 complex (Brandes et al. 2014). In addition, full recovery of the ability to generate mitoO_2_^.−^ was observed in otherwise resistant cells (e.g., acute myeloid cell lines) after supplementation with PMA, a direct NOX 2 activator (Takeya et al. 2003).

Thus, the vulnerability of APL cells to ATO induced mitoO_2_^.−^ formation, and MPT dependent apoptosis, appears to be strictly connected with their significant NOX 2 dependent ROS response.

The well-established Ca^2+^ dependence of NOX 2 activity (Brandes et al. 2014) implies that ATO increases the cytosolic concentration of the cation ([Ca^2+^]_c_) in microdomains sensed by specific Ca^2+^ dependent NOX 2 subunits. However, the source of the cation is unknown. Moreover, it is unclear whether the increased mitochondrial Ca^2+^ concentrations ([Ca^2+^]_m_) is relevant for mitoO_2_^−^. emission and for the induction of MPT, as previously observed with sodium arsenite in other cell types (NaAsO_2_) (Guidarelli et al. 2019, 2021).

Finally, although numerous reports document the ability of different concentrations of ATO to deregulate Ca^2+^ homeostasis in a variety cell types (Florea and Busselberg 2008; Florea et al. 2007; Srivastava et al. 2016; Zhang et al. 2016), we could not find systematic studies investigating the mechanisms whereby ATO mobilizes Ca^2+^ to promote Ca^2+^-dependent events in APL cells.

The present study was performed with the aim of addressing these issues in NB4 cells, comparing the effects of ATO with those mediated by NaAsO_2_, another inorganic trivalent arsenic compound. We report evidence for similar mechanisms of deregulation of Ca^2+^ homeostasis with the two arsenic compounds, characterized by an initial stimulation of the inositol 1,4,5-trisphosphate receptor (IP_3_R), and the further release of the cation from the ryanodine receptor (RyR). The fraction of Ca^2+^ derived from the IP_3_R, unlike that released from the RyR, was responsible for ATO-induced NOX 2 activation, whereas, under similar conditions, NaAsO_2_ failed to stimulate the same enzyme activity. Interestingly, the fraction of Ca^2+^ released from the RyR -unlike that released from the IP_3_R- was taken up by the mitochondria and critically mediated mitoO_2_^−^. formation with NaAsO_2_—but not with ATO -, which in fact induced mitoO_2_^.−^ formation via a Ca^2+^-independent mechanism. The induction of MPT, and of the ensuing mitochondrial apoptosis, instead required an increased [Ca^2+^]_m_ with both arsenic compounds.

## Materials and methods

### Chemicals

ATO, NaAsO_2_, rotenone, apocynin, 2-aminoethoxydiphenyl borate (2-APB), ryanodine (Ry), caffeine (Cf), ATP, phorbol-12 myristate-13-acetate (PMA), Hoechst 33342, as well as most of the reagent end chemicals, were purchased from Sigma Aldrich (Milan, Italy). Cyclosporin A (CsA) was from Novartis (Bern, Switzerland). Ru360 was from Thermo Fisher Scientific (Milan, Italy). Fluo-4- acetoxymethyl ester (AM), Rhod 2-acetoxymethyl ester (AM), MitoSOX red, and Dihydrorhodamine 123 (DHR) MitoTracker CMXRos was purchased from Molecular Probes (Leiden, The Netherlands).

ATO was dissolved in 1 N NaOH, diluted in phosphate buffer saline (PBS, 136 mM NaCl, 10 mM Na_2_HPO_4_, 1.5 mM KH_2_PO_4_, 3 mM KCl; pH 7.4), and adjusted to pH 7.4 using HCl, to generate a 10 mM solution which was further diluted in PBS to generate a 1 mM stock solution, which was kept at 4 °C for up to three days. Working solutions were freshly prepared from the stock solution by dilution in cell culture medium on the day of the experiment.

NaAsO_2_ was prepared as a 1 mM stock solution in PBS and stored at 4 °C.

Cells were exposed to ATO, NaAsO_2_ and/or other additions in complete RPMI 1640 culture medium.

### Cell cultures

NB4 cells (human pro-myelocytic leukemia), were cultured in RPMI 1640 medium (Sigma-Aldrich, Milan, Italy) supplemented with 10% fetal bovine serum (FBS, Euroclone, Celbio Biotecnologie, Milan, Italy). Culture media were supplemented with penicillin (100 units/ml) and streptomycin (100 µg/ml) (Euroclone) Cells were grown at 37 °C in T-75 tissue culture flasks (Corning Inc., Corning, NY) gassed with an atmosphere of 95% air, 5% CO_2_.

### Measurement of intracellular free calcium levels and mitochondrial Ca^2+^

NB4 cells were treated for 20 min with 4 µM Fluo 4-acetoxymethyl ester or 10 µM Rhod 2-acetoxymethyl ester and subsequently exposed for a further 10 min to ATP or Cf. In other experiments, the cells were exposed for 6 h to ATO or NaAsO_2,_ and Fluo-4-acetoxymethyl ester or Rhod 2-acetoxymethyl ester were added to the culture medium in the last 30 min of incubation.

In some experiments, the cells were exposed for 30 min with Rhod 2-acetoxymethyl ester prior to the end of the 3 h treatment with ATO, or NaAsO_2_, and subsequently treated for a further 15–10 min with PMA or Cf, respectively.

After the treatments, the cells were washed three times and fluorescence images were captured with a BX-51 microscope (Olympus, Milan, Italy), equipped with a SPOT-RT camera unit (Diagnostic Instruments, Delta Sistemi, Rome, Italy) using an Olympus LCAch 40×/0.55 objective lens. The excitation and emission wavelengths were 488 and 515 nm (Fluo 4), and 540 and 590 nm (Rhod 2) with a 5-nm slit width for both emission and excitation. Images were collected with exposure times of 100–400 ms, digitally acquired, and processed for fluorescence determination at single cell level using the ImageJ software. Mean fluorescence values were determined by averaging the fluorescence values of at least 50 cells/treatment condition/experiment.

### MitoSOX red and DHR fluorescence assay

Cells were exposed for 30 min with 5 µM MitoSOX red prior to the end of the 3–6 h treatment with ATO, or NaAsO_2_. In some experiments, the cells were exposed for 30 min with 5 µM MitoSOX red prior to the end of the 3 h treatment with ATO, or NaAsO_2_, and subsequently treated for a further 15–10 min with PMA or Cf, respectively. This fluorogenic dye allows the detection of O_2_^.−^ in the mitochondria of live cells (Mukhopadhyay et al. 2007). It is indeed readily taken up by the mitochondria via a mechanism driven by the mitochondrial membrane potential and, in these organelles, react with O_2_^.−^ generating a red signal that can be detected and quantified using fluorescence microscopy. In some experiments, MitoSOX red was replaced with 10 µM DHR, which, under the influence of various types of ROS, including hydrogen peroxide (H_2_O_2_), is oxidized in the cytosol and converted to rhodamine 123, a cationic fluorescent dye that is also accumulated in the mitochondria via a mitochondrial membrane potential-dependent mechanism. The corresponding fluorescent signal can also be detected by fluorescence microscopy and provides an estimate of ROS formation in the cytosol and mitochondrial-derived origin. Indeed, mitoO_2_^.−^ dismutates to H_2_O_2_, which eventually reaches extramitochondrial compartments and oxidizes DHR. Thus, both assays require an intact mitochondrial membrane potential. We therefore measured ROS formation under conditions in which the mitochondrial membrane potential, determined as indicated below, resulted unaffected by the specific treatments. Additional information in this direction is provided in the results section.

After the incubation with the fluorescence probes, cells were finally washed three times, and the fluorescence images were visualized using the fluorescence microscope described above.

The excitation and emission wavelengths were 488 and 525 nm (DHR) and 510 and 610 nm (MitoSOX red), with a 5-nm slit width for both emission and excitation. Images were collected with exposure times of 100–400 ms, digitally acquired, and processed for fluorescence determination at the single cell level by ImageJ software. Mean fluorescence values were determined by averaging the fluorescence values of at least 50 cells/treatment condition/experiment.

### Western blot analysis

After treatments, the cells were lysed with RIPA buffer (Thermo Fisher Scientific), with the further addition of 1 mM dithiothreitol, 10 mM Na_3_VO_4_, 10 mM NaF, 350 mM phenylmethylsulphonyl fluoride, 1% protease inhibitor complex, pH 7.5. Protein concentrations were determined with the Bradford reagent (Bio-Rad) in SPECTRA Fluor Plus Microplate Reader Tecan (Tecan, CH). The proteins were separated by polyacrylamide gel vertical electrophoresis and transferred to polyvinylidene difluoride membranes. The membranes were blocked in 5% milk and probed with primary antibodies against: phospho-p47^phox^ (Ser345) (PA5–37806, Thermo Fisher Scientific), or p47^phox^ (SAB4502810, Merk chemicals) overnight, at 4 °C. Membranes were washed 3 times for 10 min/each in Tween-Tris buffered saline and probed with secondary antibodies anti-mouse (sc-516102, Santa Cruz) or anti-rabbit (sc-2357, Santa Cruz) diluted in 5% milk Tween-Tris-buffered saline for 2 h at room temperature. p47^phox^ was used to assess the equal loading of the lanes. Membranes were visualized with ChemiDoc MP Imaging System (Bio-Rad), and relative amounts of proteins were quantified by densitometric analysis using Image J software.

### Measurement of DNA single-strand breakage by the alkaline halo assay

DNA single-strand breakage was determined using the alkaline halo assay developed in our laboratory (Cantoni and Guidarelli 2008). It is important to note that, although we refer to DNA strand scission throughout the text, the DNA nicks measured by this technique under alkaline conditions may, in fact, include alkali labile sites in addition to direct strand breaks. Details on the alkaline halo assay and processing of fluorescence images and on the calculation of the experimental results are also given in Ref. (Cantoni and Guidarelli 2008). DNA single-strand breakage was quantified by calculating the nuclear spreading factor value, representing the ratio between the area of the halo (obtained by subtracting the area of the nucleus from the total area, nucleus + halo) and that of the nucleus, from 50 to 75 randomly selected cells/experiment/treatment condition. Results are expressed as relative nuclear spreading factor values calculated by subtracting the nuclear spreading factor values of control cells from those of treated cells.

### Immunofluorescence analysis

After treatments, the cells were washed twice with PBS, suspended in 2 ml of saline A (8.182 g/l NaCl, 0.372 g/l KCl, 0.336 g/l NaHCO_3_, and 0.9 g/l glucose, pH 7.4), and incubated for 10 min in 35-mm tissue culture dishes containing an uncoated coverslip. Under these conditions, cells rapidly attach to the coverslip. Subsequently, the cells were fixed for 1 min with 95% ethanol and 5% acetic acid. Following fixation, the cells were washed with PBS. To mitigate non-specific binding, the cells were blocked in PBS containing 2% bovine serum albumin (BSA) for 30 min at room temperature. Next, the cells were incubated with monoclonal anti-cytochrome c antibodies (sc-13560, Santa Cruz), diluted 1:100 in PBS containing 2% BSA, for 18 h at 4 °C. Following antibody incubation, the cells were washed and subsequently incubated for 3 h in the dark with fluorescein isothiocyanate-conjugated secondary antibody anti-mouse (sc-516140, Santa Cruz), (diluted 1:100 in PBS). The cells were finally washed three times with PBS, and fluorescence images were taken and processed as described above. The excitation and emission wavelengths were 488 and 525 nm, with a 5-nm slit width for both emission and excitation. Images were acquired digitally and processed at the single-cell level with ImageJ software. Specifically, in the experiments assessing the mitochondrial loss of cytochrome c, the relative numbers of cells exhibiting a punctate fluorescence (indicative of its mitochondrial localization) and diffused fluorescence (suggestive of its mitochondrial loss) were counted. At least 100 cells were analyzed in each experiment to calculate the percentage of cells demonstrating a diffused fluorescence indicative of MPT-dependent mitochondrial loss of cytochrome c.

### Measurement of mitochondrial membrane potential

Cells were supplemented with 500 µM MitoTracker red CMXRos for 30 min prior to the end of the treatments. The cells were washed three times, and the fluorescence images were taken and processed as described above. The excitation and emission wavelengths were 545 and 610 nm, with a 5 nm slit width for both emission and excitation. Mean fluorescence values were determined by averaging the fluorescence values of at least 100 cells/treatment condition/experiment.

### Fluorogenic caspase 3 assays

Following the treatments, the cells were lysed with RIPA buffer containing the additions indicated above, pH 7.5. Subsequently, in a 96-well plate, 15 µg of proteins each well were loaded with caspase 3 buffer (Hepes 100 mM, Sucrose 10%, Chaps. 0.1%, EDTA 1 mM, pH 7.5) and caspase 3 substrate (Ac-DEVD-aminomethylcoumarin, 0.4 mM). Caspase 3-like activity was determined fluorometrically in SPECTRA Fluor Plus Microplate Reader Tecan (excitation at 360 nm and emission at 465 nm) by quantifying the release of aminomethylcoumarin from cleaved caspase 3 substrate.

### Analysis of apoptosis with the Hoechst 33342 assay

20 min prior to the end of the treatments, cells were incubated with 10 µM Hoechst  33342. The cells were finally analysed with a fluorescence microscope to assess their nuclear morphology (chromatin condensation and fragmentation). Cells with homogeneously stained nuclei were considered viable.

### Statistical analysis

The results are reported as means ± standard deviation (SD) and calculated from three distinct experiments. GraphPad Prism software version 9.0.0 (GraphPad Software Inc., La Jolla, CA) was used to create graphs and perform data analyses. Statistical differences were analyzed by one-way ANOVA, followed by Tukey’s test to compare every mean with every other mean or Dunnett’s test to compare every mean to a control mean. P values of < 0.05 indicated statistical significance.

## Results

### Agonist induced cytosolic and mitochondrial Ca^2+^ accumulation

**Fig. 1:**
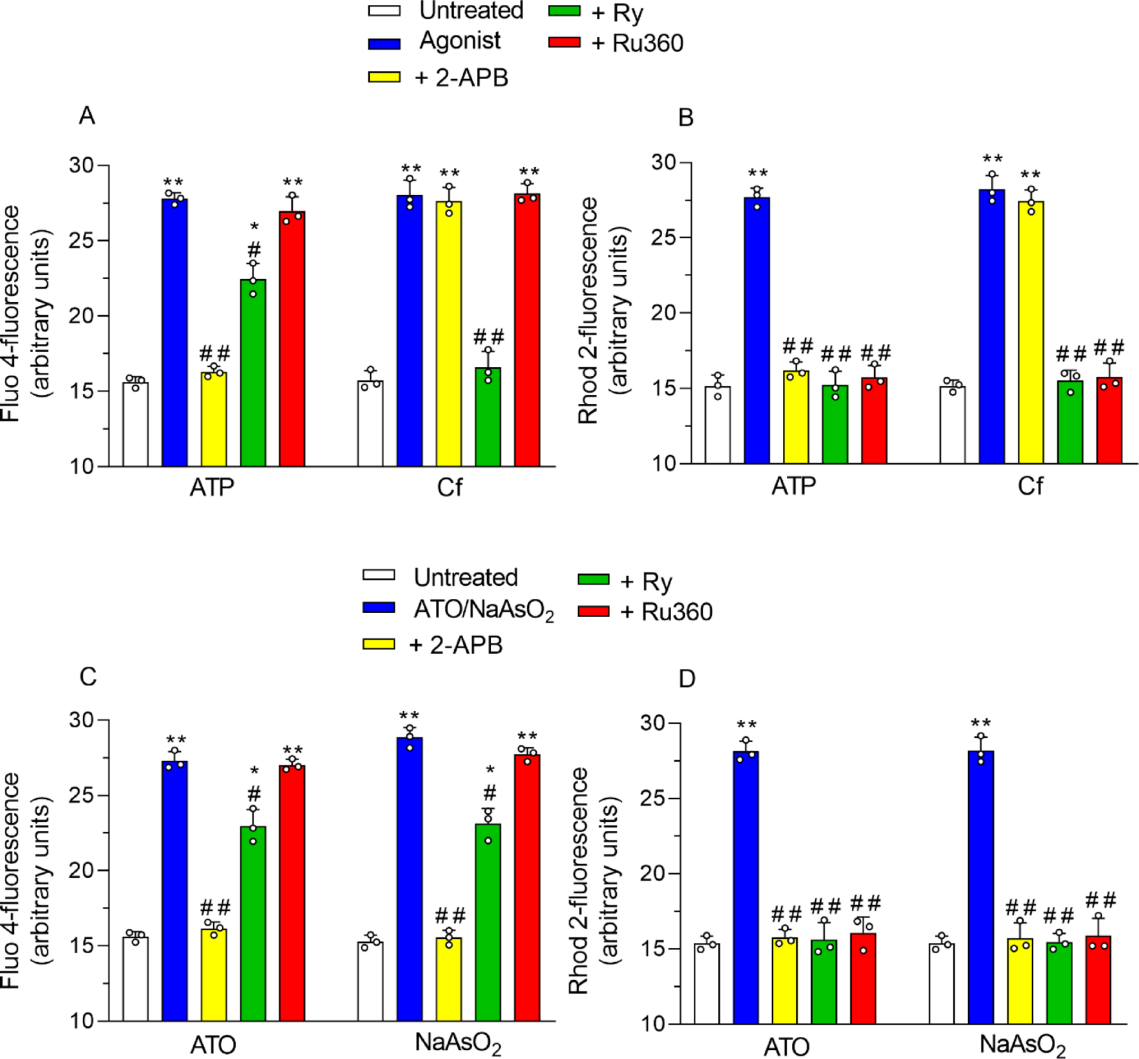
The effect of ATO and NaAsO_2_ in NB4 cell Ca^2+^ homeostasis regulation. NB4 cells were pre-exposed (20 min) to Fluo 4-AM or Rhod-2-AM and then treated for 10 min with 100 µM ATP or 10 mM Cf. 50 µM 2-APB, 20 µM Ry or 10 µM Ru360 were added to the cultures 5 min prior to ATP or Cf. In other experiments, the cells preloaded with the fluorescent probes were treated (5 min) with the vehicle, 2-APB, Ry or Ru360 and incubated for a further 6 h with either 1 µM ATO or 2.5 µM NaAsO_2_. After treatments, the cells were analyzed for Fluo 4- (**A**, **C**) or Rhod 2-(**B**, **D**) fluorescence. The results represent the means ± SD calculated from at least three distinct experiments. *P < 0.05, **P < 0.01, as compared to untreated cells. ^#^*P* < 0.05, ^##^*P* < 0.01, as compared to cells treated with ATP, Cf, ATO or NaAsO_2_ (one-way ANOVA followed by Tukey test)

NB4 cells were treated for 10 min with ATP, an agonist of GQ protein coupled purinergic receptors (Berridge 2016), to stimulate Ca^2+^ release from the IP_3_R. Based on concentration-dependence studies (Suppl. Figure 1 A), ATP was used at 100 µM, causing maximal elevation of the [Ca^2+^]_c_, detected with the fluorescent probe Fluo 4 (Gee et al. 2000). Under these conditions, ATP increased the cytosolic Ca^2+^ signal via a mechanism suppressed by 50 µM 2-APB, an IP_3_R antagonist (Berridge 2016), significantly reduced by 20 µM Ry, a RyR antagonist (Meissner 2017), and unaffected by 10 µM Ru360, a mitochondrial Ca^2+^ uniporter (MCU) inhibitor (Zazueta et al. 1999) (Fig. 1A). The concentration of Cf (10 mM, 10 min), a RyR agonist (Meissner 2017), was also selected after concentration dependence studies (Suppl. Figure 1B), and caused an increase in [Ca^2+^]_c_ insensitive to 2-APB, or Ru360, and completely suppressed by Ry (Fig. 1A).

Other experiments were next performed to detect the accumulation of mitochondria Ca^2+^ using the fluorescence probe Rhod 2. 100 µM ATP increased significantly the [Ca^2+^]_m_ via a mechanism suppressed by 2-APB as well as by Ry or Ru360 (Fig. 1B). The increase in [Ca^2+^]_m_ induced by 10 mM Cf was instead insensitive to 2-APB and suppressed by Ry or Ru360 (Fig. 1B).

These results therefore indicate that in NB4 cells, as we previously observed in other cell types (Guidarelli et al. 2021), ATP causes Ca^2+^ mobilization from the IP_3_R, thereby leading to further Ca^2+^ release from the RyR. These events were then associated with the mitochondrial accumulation of Ca^2+^ which was entirely derived from the RyR. These experiments, as well as those using Cf in the place of ATP, also provide an indication on the specificity of the effects mediated by the antagonists and inhibitors employed.

### The effects of ATO on cytosolic and mitochondrial Ca^2+^ homeostasis

We investigated the effects of a 6 h exposure of NB4 cells to a clinically relevant (Westervelt et al. 2001; Zhang et al. 2010) concentration of ATO (1 µM) on the [Ca^2+^]_c_. Under these conditions there were no signs of toxicity, as detected by visual inspection of the cultures or by measuring apoptotic DNA fragmentation, instead clearly detected at later time intervals (Suppl. Figure 2).

It was then interesting to observe that the Ca^2+^ response induced under these conditions was completely, or only partially, reduced by 2-APB or Ry (Fig. 1C), respectively. Instead, Ru360 was ineffective, as expected. Qualitatively identical results were obtained in experiments in which ATO was replaced with 2.5 µM NaAsO_2_ (Fig. 1C), which is in keeping with our previous results obtained in other cell lines (Guidarelli et al. 2019, 2021).

These findings were next extended by showing that, under the same conditions, the two arsenic compounds increase the [Ca^2+^]_m_ via mechanisms suppressed by 2-APB, Ry and Ru360 (Fig. 1D).

The above findings indicate that ATO and NaAsO_2_ similarly affect Ca^2+^ homeostasis, recapitulating the effects previously observed with ATP. Thus, the two arsenic compounds initially target the IP_3_R to then stimulate further Ca^2+^ release from the RyR. The fraction of the cation released by the RyR, unlike that derived from the IP_3_R, is then taken up by the mitochondria.

### IP_3_R derived Ca^2+^ regulates NOX 2 activation and the ensuing ROS formation induced by ATO

We addressed the issue of the Ca^2+^ dependent regulation of NOX 2 activity in cells exposed to ATO, and for this purpose measured the phosphorylation of the p47^phox^ subunit, an essential component of the NOX 2 complex. It is indeed well established that p47^phox^ phosphorylation is crucial for the assembly and activation of the oxidase complex, thereby providing a good indication of NOX 2 activation (Brandes et al. 2014).

**Fig. 2:**
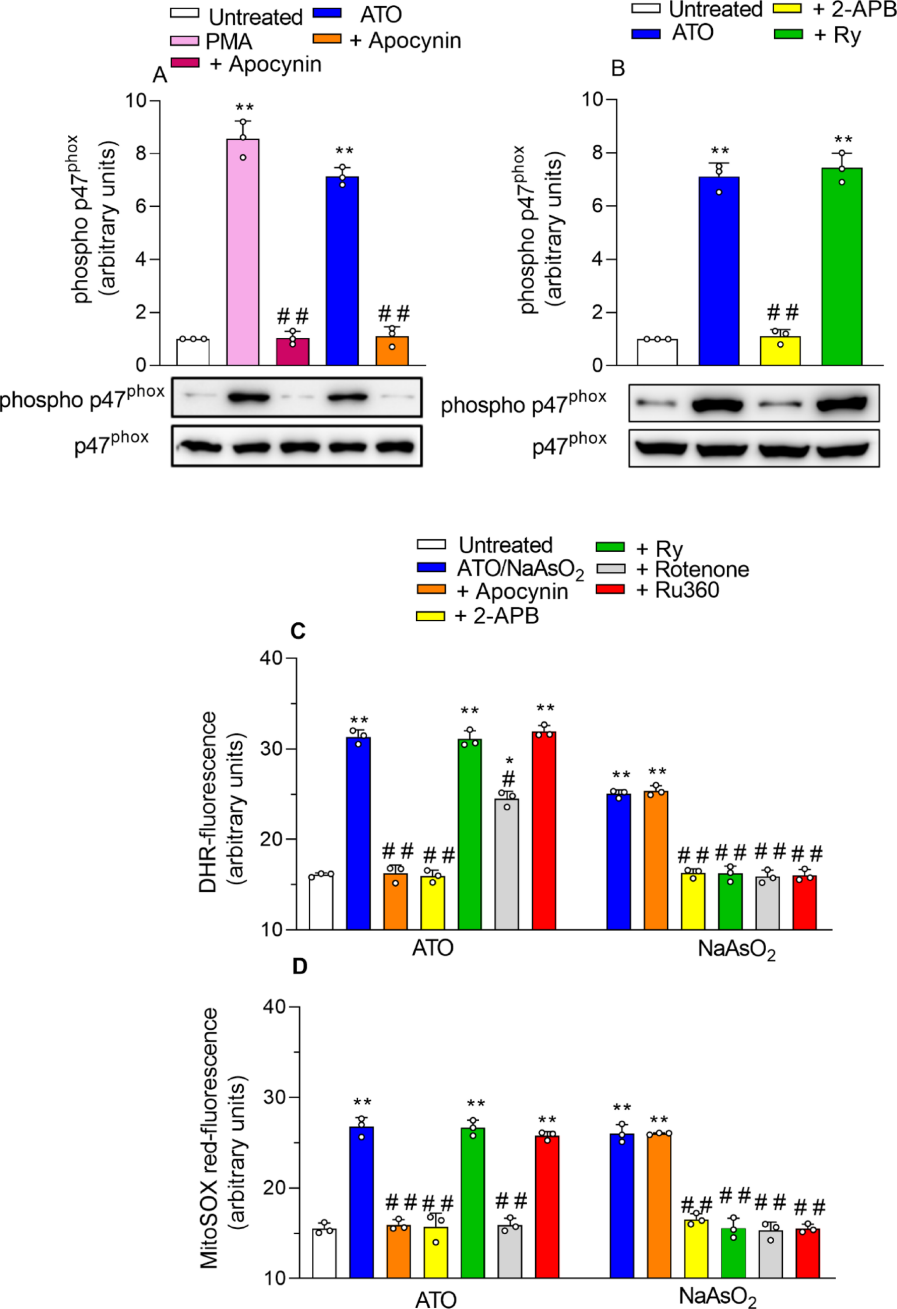
IP_3_R-derived Ca^2+^ mediates NOX 2 activation and ROS formation induced by ATO. NB4 cells were pretreated (5 min) with the vehicle, or 10 µM apocynin and then exposed for 15 min to 0.162 µM PMA or for 6 h to 1 µM ATO. After treatments, the cells were analyzed for phospho p47^phox^ expression (**A**). In other experiments, the cells were pretreated with the vehicle, 2-APB or Ry, incubated for 6 h with 1 µM ATO and finally analyzed for phosphor p47^phox^ expression (**B**). The relative band intensity of phospho p47^phox^ is depicted in the top bar chart of A and B. p47^phox^ was accounted for loading control. In the final set of experiments, the cells were pretreated with the vehicle, apocynin, 2-APB, Ry, 0.5 µM rotenone or Ru360, incubated for 6 h with 1 µM ATO, or 2.5 µM NaAsO_2_, and finally analyzed for DHR- (**C**) or MitoSOX red- (**D**) fluorescence. Results represent the means ± SD calculated from three separate experiments. *P < 0.05, **P < 0.01, compared with untreated cells. #*P* < 0.05, ##*P* < 0.01 compared with PMA, ATO or NaAsO_2_ treated cells (one-way ANOVA followed by Tukey test)

We determined that PMA (15 min; 0.162 µM) as well as 1 µM ATO (6 h) increase p47^phox^ phosphorylation via mechanisms sensitive to the NOX 2 inhibitor apocynin (10 µM; (Brandes et al. 2014) (Fig. 2A). Most importantly, p47^phox^ phosphorylation induced by ATO was suppressed by 2-APB and insensitive to Ry (Fig. 2B).

As we recently reported, 2.5 µM NaAsO_2_ fails to stimulate p47^phox^ phosphorylation (Spina et al. 2025). Consistently, there was no evidence of this response in cells exposed to NaAsO_2_, instead detected with the positive control PMA (Supp. Figure 3).

**Fig. 3:**
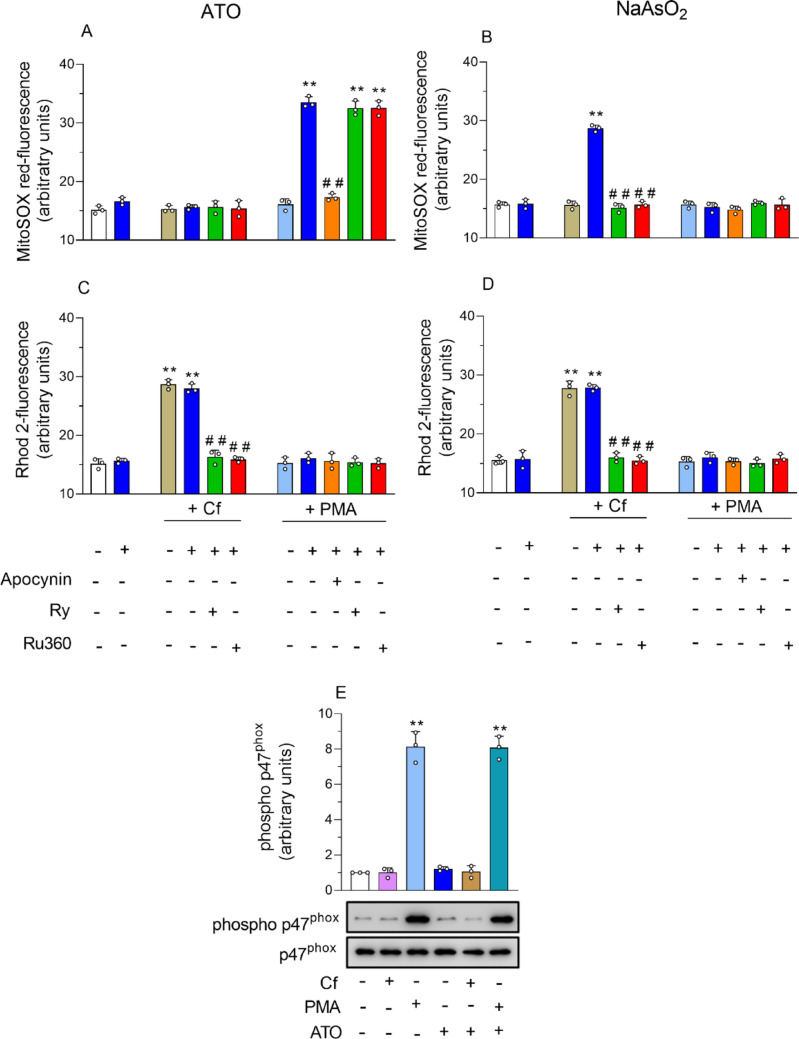
ATO-induced mitochondrial ROS formation is upstream NOX 2 dependent and does not require an increased mitochondrial Ca^2+^ concentration. NB4 cells were treated for 3 h with the vehicle, 1 µM ATO (**A**, **C**), or 2.5 µM NaAsO_2_ (**B**, **D**), and supplemented in the last 15 and 10 min of incubation with PMA (0.162 µM) or Cf (10 mM), respectively. In some experiments, Ry, Ru360, or apocynin, was added to the cultures 5 min prior to PMA, or Cf. After treatments, the cells were analyzed for MitoSOX red- (**A**, **B**), or Rhod 2- (**C**, **D**) fluorescence. In other experiments, the cells were treated for 3 h with the vehicle, or 1 µM ATO, and PMA or Cf added during the last 15 and 10 min of incubation, respectively. After treatments, the cells were analyzed for phospho p47^phox^ expression (**E**). The relative band intensity of phospho p47^phox^ is depicted in the top bar chart. p47^phox^ was accounted as loading control The results represent the means ± SD calculated from three distinct experiments. **P < 0.01, as compared to untreated cells. ^##^*P* < 0.01, as compared to cells treated with ATO or NaAsO_2_ (one-way ANOVA followed by Tukey test)

Thus, in NB4 cells, IP_3_R derived Ca^2+^ mediates NOX 2 activation induced by ATO. There was instead no apparent role for the fraction of the cation released from the RyR.

We then measured ROS formation using the fluorescent probe DHR, which reveals the presence of various types of cytosolic ROS, including NOX 2 and mitochondrially derived H_2_O_2_ (Gomes et al. 2005).

As illustrated in Fig. 2C, 1 µM ATO induced a significant DHR fluorescence response, sensitive to apocynin and to 2-APB, and insensitive to Ry. In addition, the DHR fluorescence response induced by ATO was partially reduced by the complex I inhibitor rotenone (0.5 µM, (Degli Esposti 1998) and insensitive to Ru360.

The results obtained with NaAsO_2_ were significantly different. We indeed observed that the resulting DHR fluorescence response was insensitive to apocynin and suppressed by 2-APB, Ry, Ru360 and rotenone (Fig. 2C). These results are therefore in keeping with the outcomes of previous studies using other cell lines (Guidarelli et al. 2019, 2021) indicating that, under these conditions, the DHR fluorescence response is uniquely mediated by mitochondrially derived H_2_O_2_. This explains insensitivity to apocynin and complete suppression by rotenone. Moreover, as will be discussed below, mitoO_2_^.−^ emission induced by NaAsO_2_ is regulated by the increased [Ca^2+^]_m_ (Guidarelli et al. 2019, 2021), which is in keeping with the observed suppressive effects mediated by Ry or Ru360 (Fig. 2C).

The outcome of the above experiments obtained with ATO is instead of more complex interpretation. The observation that apocynin suppresses ROS formation documents the critical role of NOX 2 in inducing these responses, both directly and indirectly (i.e., by triggering downstream mitoO_2_^.−^ emission, (Spina et al. 2025). Partial inhibition of ROS formation by rotenone implies the contribution of mitochondrially derived H_2_O_2_ in the induction of the overall DHR fluorescence response. Sensitivity to 2-APB is instead readily explained by the notion that IP_3_R released Ca^2+^ is required for NOX 2 activation (Fig. 2B). Finally, insensitivity to Ry, or Ru360, strongly suggests that ATO induced mitoO_2_^.−^ emission is Ca^2+^ independent. In other words, the increased [Ca^2+^]_m_ induced by ATO bears no consequences in the events associated with mitoO_2_^.−^ formation.

Collectively, these results indicate that ATO triggers NOX 2 activation and NOX 2 dependent ROS formation via a mechanism uniquely regulated by the fraction of Ca^2+^ released by the IP_3_R. IP_3_R-derived Ca^2+^ is then connected with the induction of further Ca^2+^ release from the RyR, which, unlike the IP_3_R derived Ca^2+^, is cleared by the mitochondria. However, ATO caused mitoO_2_^.−^ emission via an apparently Ca^2+^- independent mechanism (more evidence is presented below), strictly controlled by the upstream NOX 2 dependent ROS formation (Spina et al. 2025). NaAsO_2_ also caused IP_3_R and downstream RyR activation coupled with mitochondrial Ca^2+^ accumulation, although, under these conditions, mitoO_2_^.−^ formation occurred in the absence of detectable NOX 2 activation and was critically dependent on the increased [Ca^2+^]_m_.

### ATO induces mitochondrial ROS formation via a mechanism that does not require mitochondrial Ca^2+^ accumulation

To more directly address the issue of the Ca^2+^ dependence of mitoO_2_^.−^ formation, we moved to experiments using MitoSOX red, a fluorescence probe specifically detecting the formation of these radicals in live cells (Mukhopadhyay et al. 2007). We found that ATO induces a significant MitoSOX red fluorescence response, suppressed by apocynin, 2-APB or rotenone, and unaffected by Ry or Ru360 (Fig. 2D), thereby implying that conditions associated with NOX 2 activation are permissive for mitoO_2_^.−^ formation also in the absence of increased [Ca^2+^]_m_.

The MitoSOX red fluorescence response evoked by NaAsO_2_ was instead suppressed by 2-APB, Ry, Ru360 or rotenone, and unaffected by apocynin. This observation emphasizes the pivotal role of [Ca^2+^]_m_ in events associated with NaAsO_2_-induced mitoO_2_^.−^ formation, occurring in the absence of concomitant NOX 2 activation (Supp. Figure 3) (Spina et al. 2025).

Thus, these results are in keeping with those previously obtained with DHR, thereby putting more weight on the notion that mitoO_2_^−^. formation is Ca^2+^-dependent when driven by NaAsO_2_ -but not by ATO.

To further address this issue, we performed experiments in which the cells were treated for 3 h with ATO, or NaAsO_2_, i.e., conditions associated with undetectable mitoO_2_^.−^ formation (Fig. 3A and B). We then repeated these experiments with the addition of Cf in the last 10 min of the 3 h incubation. As indicated in Fig. 3, under these conditions, ATO (C) and NaAsO_2_ (D) failed to enhance the [Ca^2+^]_m_, which was however significantly increased after the additional supplementation of Cf, via a mechanism suppressed by both Ry and Ru360.

We then observed that the use of Cf to elevate the [Ca^2+^]_m_ was not accompanied by mitoO_2_^.−^ emission in cells exposed to ATO (Fig. 3A). In remarkable contrast, Cf supplementation was associated with significant mitoO_2_^.−^ emission in cells exposed to NaAsO_2_ (Fig. 3B). The observation that the enhancing effects of Cf were due to the increased [Ca^2+^]_m_ is in keeping with the suppressive effects mediated by Ry or Ru360 (Fig. 3B).

The fact that the 3 h exposure to NaAsO_2_ fails to elicit mitoO_2_^.−^ formation is therefore likely dependent on lack/insufficient [Ca^2+^]_m_ and responsiveness is therefore restored by Cf, which releases Ca^2+^ from the RyR, an event associated with the mitochondrial clearance of the cation.

However, ATO fails to induce significant NOX 2 activation (Fig. 3E), due to low Ca^2+^ release from the IP_3_R, and addition of Cf fails to increase NOX 2 activity (Fig. 3E). This is an expected finding, in keeping with the notion that RyR derived Ca^2+^ is not sensed in microdomains in which the Ca^2+^ sensitive NOX 2 isoforms are compartmentalized (Fig. 2B). Thus, ATO failed to promote mitoO_2_^−^. formation under conditions in which the [Ca^2+^]_m_ was increased.

Different results were instead obtained by replacing Cf with PMA, which promoted p47phox phosphorylation (Fig. 3E), and restored ATO-induced mitoO_2_^.−^ emission (Fig. 3A). Importantly, this response was insensitive to Ry, or Ru360, and suppressed by apocynin, as previously observed using the 6 h exposure protocol (Fig. 2D). In addition, PMA alone, or associated with ATO, failed to increase the [Ca^2+^]_m_ (Fig. 3C). As a final note, PMA failed to affect the [Ca^2+^]_m_ (Fig. 3D) and promote mitoO_2_^.−^ formation (Fig. 3B) in cells treated with NaAsO_2_. These findings are therefore in keeping with the notion that NOX 2 activation is a critical upstream event in ATO induced mitoO_2_^.−^ formation, with no detectable role in the same response induced by NaAsO_2_. In addition, rescue experiments using PMA once again demonstrate the occurrence of ATO induced mitoO_2_^.−^ formation in the absence of increased [Ca^2+^]_m_.

The results presented in this section collectively demonstrate that the mitochondrial events regulating O_2_^−^. formation are Ca^2+^-independent. This mechanism is therefore remarkably different from that observed with NaAsO_2_, which does not require concomitant NOX 2 activation and is critically regulated by the [Ca^2+^]_m_. Since the fraction of Ca^2+^ cleared by the mitochondria derives from the RyR with both arsenic compounds, the above results indicate that this Ca^2+^ pool is of pivotal importance for mitoO_2_^.−^ emission induced by NaAsO_2_, with hardly any role in the regulation of the same response elicited by ATO.

### Role of NOX2 and mitochondrial superoxide in the downstream induction of strand scission of genomic DNA mediated by ATO

**Fig. 4:**
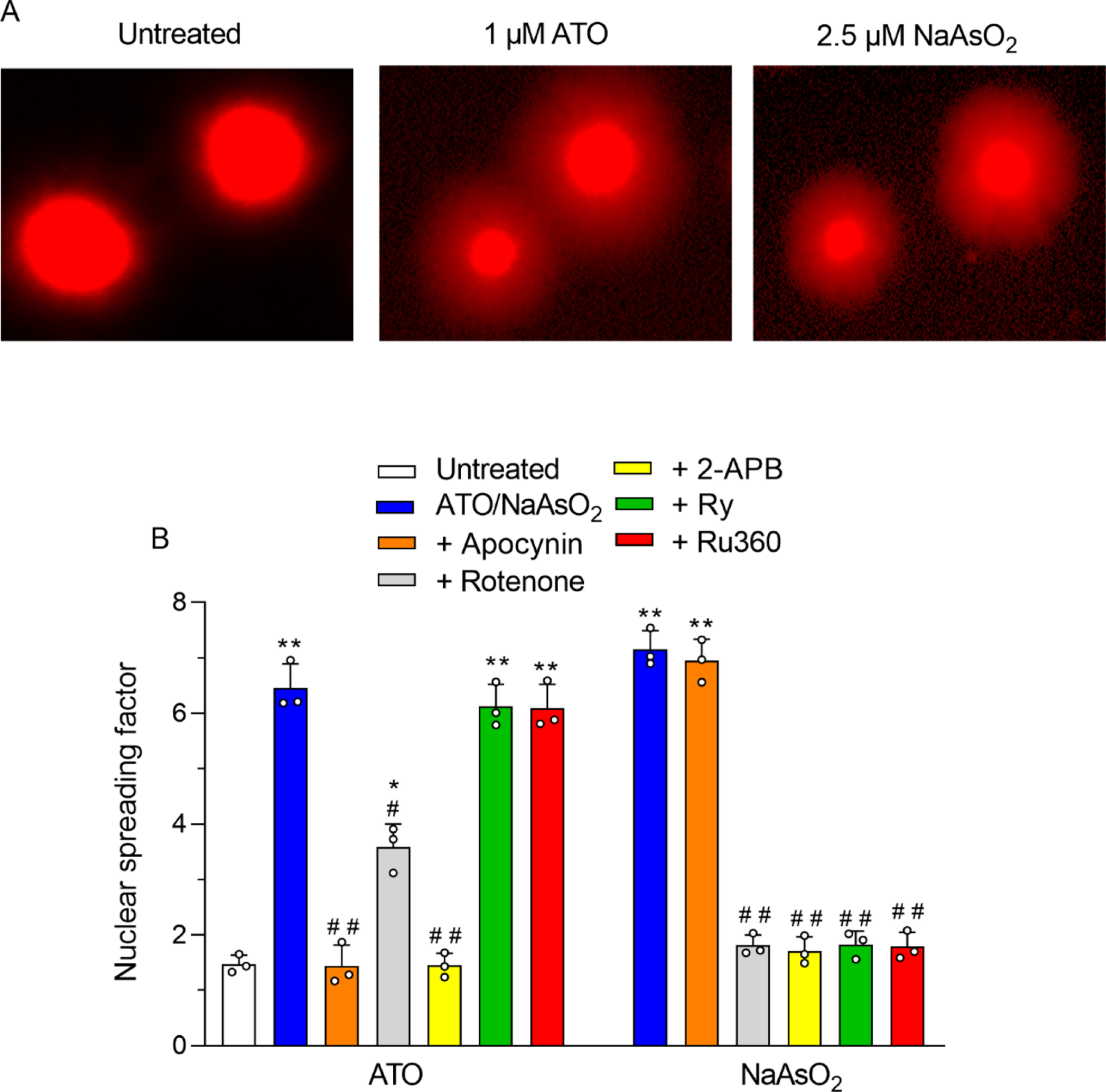
DNA strand breaks induced by ATO are mediated by both mitochondrially and NOX 2 derived ROS. (**A**) Representative fluorescent photomicrographs of U937 cells exposed for 6 h to 1 µM ATO, or 2.5 µM NaAsO_2_, and subsequently processed with the alkaline halo assay. (**B**) NB4 cells were pretreated for 5 min with the vehicle, apocynin, rotenone, 2-APB, Ry or Ru360 and incubated for a further 6 h with 1 µM ATO, or 2.5 µM NaAsO_2_. The level of DNA strand breaks was measured immediately after treatments using the alkaline halo assay. The results represent the means ± SD calculated from three distinct experiments. *P < 0.05, **P < 0.01, as compared to untreated cells. ^#^*P* < 0.05, ^##^*P* < 0.01, as compared to cells treated with ATO or NaAsO_2_ (one-way ANOVA followed by Tukey test)

NB4 cells were exposed for 6 h to 1 µM ATO and analyzed with the alkaline halo assay, a sensitive technique that measures DNA strand breaks/alkali labile sites at the single cell level (Cantoni and Guidarelli 2008). Visual inspection of ethidium bromide-stained untreated and treated cells revealed significant differences (Fig. 4A), indicative of the formation of the above lesions. Indeed, ATO increased the size of the halos and caused a parallel reduction of the size of the nuclear remnants, an effect that was quantified by counting 70–90 cells/treatment condition in three separate experiments (Fig. 4B). Next, we determined that the DNA damaging response induced by ATO is suppressed by apocynin and 2-APB, unaffected by Ry and Ru360, and significantly reduced by the complex I inhibitor rotenone.

Evidence of DNA strand scission was also obtained with NaAsO_2_ (Fig. 4A and B) and this response was insensitive to apocynin and suppressed by 2-APB, Ry, Ru360 and rotenone.

These results indicate that a large proportion of the strand scission of genomic DNA induced by ATO is generated by species of mitochondrial origins, i.e., H_2_O_2_ derived from mitoO_2_^.−^, a conclusion consistent with the significant inhibitory effects of rotenone. The rotenone resistant fraction of the DNA lesions is mediated by NOX 2 derived ROS. DNA strand scission generated by NaAsO_2_ was instead entirely dependent on the Ca^2+^-dependent mitochondrial formation of mitoO_2_^.−^ and the ensuing formation of diffusible H_2_O_2_.

### RyR derived mitochondrial Ca^2+^ critically mediates mitochondrial permeability transition- dependent apoptosis induced by ATO

In the last part of this study, we investigated the role of mitochondrial Ca^2+^ in events associated with ATO induced MPT-dependent apoptosis.

**Fig. 5:**
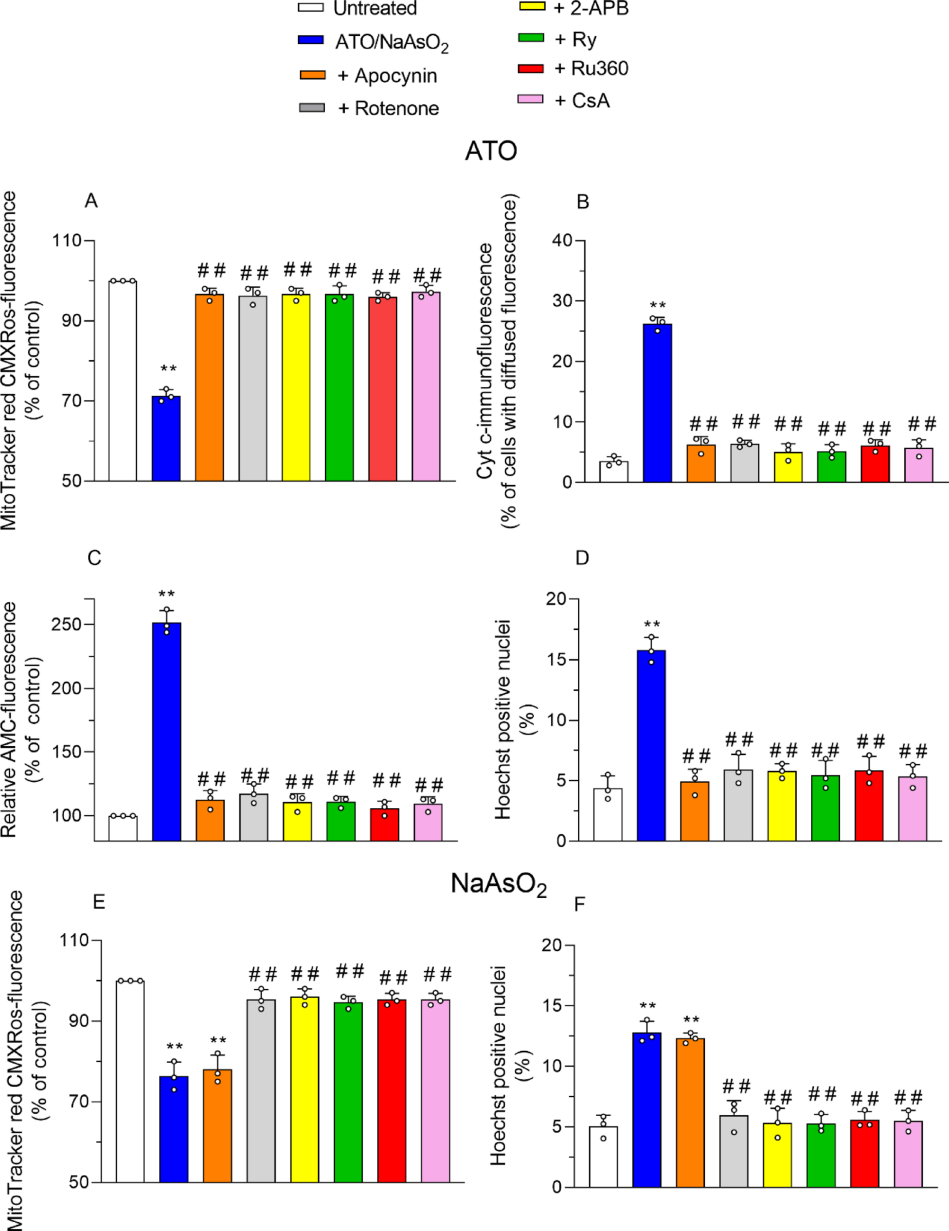
Mitochondrial permeability transition and the ensuing apoptosis induced by ATO requires RyR-mediated mitochondrial Ca^2+^ uptake. NB4 cells were pretreated for 5 min with vehicle, apocynin, rotenone, 2-APB, Ry, Ru360, or 0.5 µM CsA and incubated for a further 9 (**A**, **B**, **E**) or 14 (**C**, **D**, **F**) h with 1 µM ATO (**A**-**D**), or 2.5 µM NaAsO_2_ (**E**, **F**). After treatments, the cells were analyzed for MitoTracker red CMXRos-fluorescence (**A**, **E**), cytochrome c (Cyt c) subcellular localization (**B**), caspase 3 activity (**C**) and apoptotic DNA fragmentation/condensation (**D**, **F**). The results represent the means ± SD calculated from three distinct experiments. **P < 0.01, as compared to untreated cells. ^##^*P* < 0.01, as compared to cells treated with ATO or NaAsO_2_ (one-way ANOVA followed by Tukey test)

A 9 h exposure of NB4 cells to 1 µM ATO caused a decline in mitochondrial membrane potential (Fig. 5A) and the mitochondrial loss of cytochrome c (Fig. 5B). Both events were prevented by apocynin, rotenone or 0.5 µM CsA, a MPT inhibitor (Halestrap et al. 1997), consistently with our previous findings indicating that ATO induces MPT via a mechanism connected with mitoO_2_^.−^ emission downstream to NOX 2 activation (Spina et al. 2025). We implemented these findings by showing that the decline in mitochondrial membrane potential, and the mitochondrial loss of cytochrome c, were also suppressed by 2-APB, Ry and Ru360 (Fig. 5B). In other experiments, ATO-induced caspase 3 activation (Fig. 5C) and apoptotic DNA fragmentation (Fig. 5D) were monitored at 14 h, and both responses were suppressed by all the above inhibitors.

Thus, 2-APB, Ry and Ru360, by intercepting Ca^2+^ signaling at various levels converging in downstream inhibition of mitochondrial Ca^2+^ accumulation (Fig. 1D), prevent the decline in mitochondrial potential (Fig. 5A) associated with the MPT-dependent apoptotic signaling (Fig. 5D). These findings therefore implicate RyR derived Ca^2+^ in the induction of ATO dependent MPT and in the ensuing mitochondrial pathway of apoptosis. The IP_3_R dependent Ca^2+^ release, besides being involved in NOX 2 activation, indirectly regulates these events by promoting the release of the cation from the RyR.

Parallel experiments were performed in NB4 cells exposed for 9 h to 2.5 µM NaAsO_2_, i.e., a condition associated with a decline in mitochondrial membrane potential sensitive to rotenone 2-APB, Ry, Ru360 and CsA (Fig. 5E). Apocynin instead failed to promote detectable protective effects. In addition, NaAsO_2_ was a poor inducer of apoptotic DNA fragmentation/condensation, at 14 h, which was characterized by the same inhibitor sensitivity observed with ATO (Fig. 5F). These results are in keeping with our previous findings obtained in other cell lines, indicating that RyR derived Ca^2+^ is critically connected with the triggering of MPT-dependent apoptosis induced by NaAsO_2_ (Guidarelli et al. 2019, 2021).

## Discussion

This study provides novel mechanistic insights into a cascade of events triggered by a clinically relevant concentration of ATO in NB4 cells, namely deregulation of Ca^2+^ homeostasis, Ca^2+^-dependent activation of NOX 2 and downstream mitochondrial ROS formation, induction of DNA single-strand breakage and delayed mitochondrial apoptosis.

We found that the Ca^2+^ response evoked by ATO (1 µM) is remarkably like that observed with 2.5 µM NaAsO_2_, or after a short exposure to the purinergic receptor agonist ATP. Thus, with each of the three agents, there was an initial mobilization of Ca^2+^ from the IP_3_R, and a further release of the cation from the RyR which, unlike the Ca^2+^ released from the IP_3_R, was cleared by the mitochondria. Cf, a *bona fide* RyR agonist, also directly caused rapid mitochondrial Ca²⁺ accumulation.

These results indicate that in NB4 cells, as previously observed in other cell types (Guidarelli et al. 2021), Ca^2+^ signaling events initiated by IP_3_R stimulation cause the mobilization of the cation from the RyR, promptly cleared by the mitochondria. This is consistent with the existence of close contacts sites between the RyR and mitochondria in NB4 cells. In addition, the identical responses obtained with ATO and NaAsO_2_ are consistent with the possibility that trivalent arsenic binds to selected protein thiols of the IP_3_R and cause its activation.

It is important to note that there are multiple reasons for using both arsenic compounds in our study. In addition to enabling the identification of key similarities, such as the regulation of Ca^2+^ homeostasis, this approach also helps reveal differences linked to yet undefined steps in the mechanisms of action of ATO in sensitive NB4 cells. In this context, notable examples include the Ca^2+^-dependent regulation of NOX 2 and mitochondrial ROS production. As a final note, parallel experiments with NaAsO_2_ and ATO were essential for clarifying the specificity of the effects of the inhibitors.

It is well established that, under the above treatment conditions, ATO, unlike NaAsO_2_, stimulates the activity of NOX 2, a Ca^2+^-dependent enzyme (Brandes et al. 2014). We found that p47^phox^ phosphorylation and the ensuing NOX 2 dependent ROS formation were entirely regulated by IP_3_R derived Ca^2+^. In contrast, RyR-derived Ca^2+^, while robustly triggered by ATO, did not contribute to NOX 2 activation.

These results therefore imply that Ca^2+^ sensitive NOX 2 subunits are localised in subcellular microdomains in which stimulation of the IP_3_R leads to high local [Ca^2+^] allowing assembly and activation of the NOX 2 complex.

We also found significant differences in the mechanisms whereby ATO and NaAsO_2_ promote the formation of mitoO_2_^.−^. The results obtained with NaAsO_2_ in NB4 cells were in line with those from experiments using other RyR expressing cell types (Guidarelli et al. 2021). Thus, mitoO_2_^.−^ emission occurred in the absence of NOX 2 activation and was suppressed by inhibition of Ca^2+^ mobilization from the IP_3_R, RyR and MCU-dependent Ca^2+^ transport. This indicates that NaAsO_2_, under the conditions employed in our experiments, produces mitoO_2_^.−^ via a Ca^2+^-dependent mechanism, in the absence, and hence independently, of NOX 2 activation.

In remarkable contrast, ATO induced NOX 2 activation was critically connected with downstream mitoO_2_^.−^ production (Spina et al. 2025). Since NOX 2 activation is driven by IP_3_R released Ca^2+^, NOX 2 inhibition or IP_3_R antagonism, also suppressed the mitochondrial mechanism of ROS formation. In contrast, inhibition of Ca^2+^ mobilization from the RyR, or suppression of MCU-dependent Ca^2+^ transport, had no impact on mitoO_2_^.−^ emission. Thus, these findings delineate a NOX 2-driven, Ca^2+^-independent mitochondrial mechanism for ATO induced mitoO_2_^.−^ production.

We used a second approach to support these findings, and more specifically treated the cells for 3 h with each of the two arsenic compounds, with or without the addition of either Cf or PMA. Under these conditions ATO, or NaAsO_2_, failed to enhance the [Ca^2+^]_m_ or to increase NOX 2 activity. Instead, these events were readily detected after supplementation of Cf or PMA, respectively. We found that mitoO_2_^.−^ production was restored by Cf in cells treated with NaAsO_2_, with hardly any effect being detected in cells treated with ATO. Instead, PMA elicited mitoO_2_^.−^ emission in cells exposed to ATO, in the absence of detectable effects induced by NaAsO_2_.

Thus, the results thus far presented indicate that ATO and NaAsO_2_ promote similar early effects on Ca^2+^ homeostasis and remarkably different downstream mechanisms of ROS formation. On the one hand, ATO stimulation of NOX 2 activity was uniquely based on IP_3_R derived Ca^2+^ and critically connected with the triggering of a Ca^2+^ independent mechanism of mitoO_2_^.−^ emission. On the other hand, NaAsO_2_ induced Ca^2+^ release from the IP_3_R failed to promote NOX 2 activation but was nevertheless critical for Ca^2+^-dependent triggering of mitoO_2_^.−^ formation. More specifically, Ca^2+^ release from the IP_3_R was associated with further Ca^2+^ release from the RyR, followed by the mitochondrial uptake of the cation.

MitoO_2_^.−^, regardless of the mechanism of its production, is expected to dismutate to diffusible H_2_O_2_ and mediate similar extramitochondrial effects. For this purpose, we monitored early strand scission of genomic DNA, which is not representative of apoptotic DNA fragmentation since this event occurs at later times. DNA damage detected after the 6 h exposure to NaAsO_2_ was suppressed by rotenone as well as by inhibition of Ca^2+^ signaling at the level of the IP_3_R, RyR and MCU transport. These results mirror those obtained in experiments measuring the Ca^2+^ dependent regulation of mitoO_2_^.−^ emission and are therefore in keeping with the existence of a causal link between these two events. Thus, NaAsO_2_ generates DNA strand scission via a mechanism involving mitoO_2_^.−^ formation and dismutation to H_2_O_2_, which can then diffuse to the nuclear compartment and site specifically cause DNA damage via the Fenton reaction (Winterbourn 2008).

In the case of ATO, mitochondrially derived ROS contributed to DNA strand scission, in addition to NOX 2-derived ROS, with rotenone-sensitive mitochondrial ROS playing a predominant role.

Importantly, both ATO and NaAsO_2_, while relying on different mechanisms of mitoO_2_^.−^ formation, converged on the Ca^2+^-dependent induction of MPT to then trigger the mitochondrial pathway of apoptosis.

**Fig. 6:**
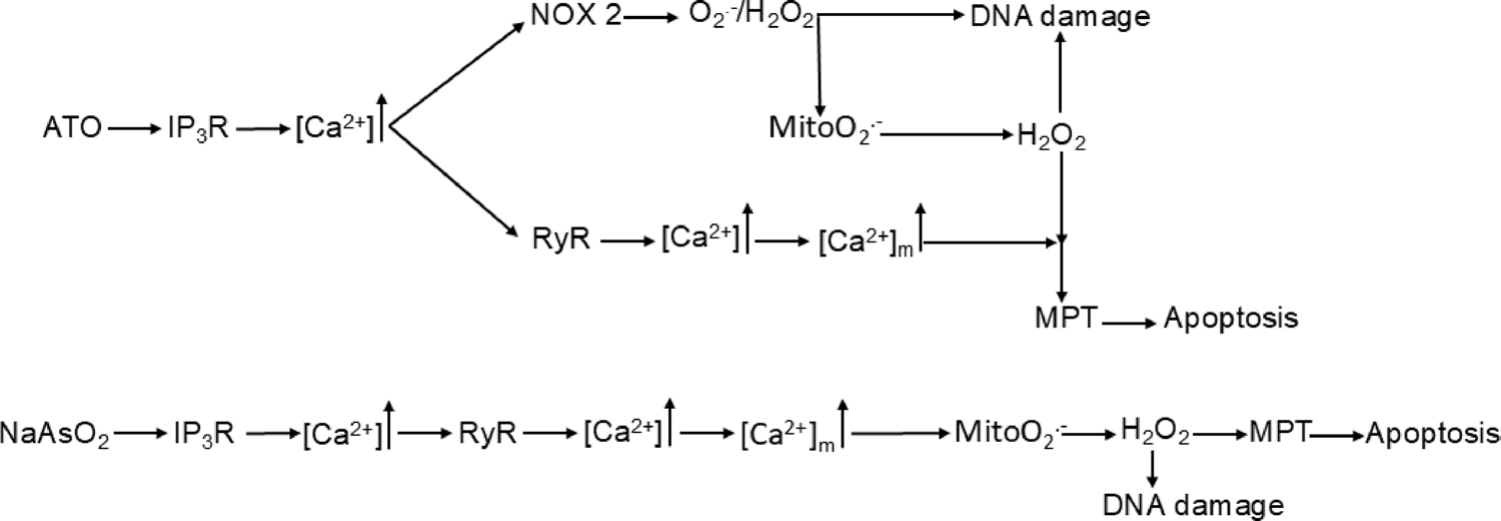
Schematic representation of the sequence of events leading to deregulation of Ca^2+^ homeostasis and Ca^2+^-dependent NOX 2 activation, mitoO_2_^.−^ production, DNA damage, and mitochondrial permeability transition dependent apoptosis induced by ATO or NaAsO_2_ in NB4 cells. The scheme illustrates two mechanistic pathways through which ATO and NaAsO_2_ disrupt redox balance and Ca^2+^ homeostasis, leading to ROS generation and other downstream cellular events. ATO promotes IP_3_R-mediated Ca^2+^-dependent activation of NOX 2, resulting in Ca^2+^-independent mitoO_2_^.−^ production. In contrast, NaAsO_2_ induces mitoO_2_^.−^ formation via an IP_3_R/RyR/mitochondrial Ca^2+^-driven mechanism. Regardless of the specific pathway of mitoO_2_^.−^ generation, the resulting H_2_O_2_ causes early DNA strand breaks (further amplified by NOX 2-derived H_2_O_2_ in the case of ATO), and triggers MPT-dependent apoptosis, which is tightly regulated by RyR-mediated Ca^2+^ release with both arsenic compounds

Collectively, these findings delineate two mechanistic routes (Fig. 6) by which the two trivalent arsenic compounds perturb Ca^2+^ and redox homeostasis: (i) an IP_3_R Ca^2+^ dependent NOX 2 activation associated with a NOX 2-driven, Ca^2+^-independent mitochondrial ROS pathway in the case of ATO, and (ii) a IP_3_R/RyR/mitochondrial Ca^2+^-driven pathway with NaAsO_2_. Regardless of the mechanism of mitoO_2_^.−^ formation, the resulting H_2_O_2_ caused early DNA strand scission (integrated by NOX 2 derived H_2_O_2_ in the case of ATO), and MPT-dependent apoptosis.

This duality may underlie both the therapeutic efficacy of ATO in APL and the toxicological liabilities associated with arsenic exposure. Future work should address whether these mechanistic differences translate into distinct cellular stress responses in non-leukemic tissues, potentially explaining the divergent clinical outcomes associated with arsenic exposure and/or therapy. The distinct reliance on NOX 2 versus mitochondrial Ca^2+^-dependent pathways for ROS production and apoptosis offers potential targets for selectively modulating treatment responses and minimizing ATO toxicity in leukemia therapy.

## Supplementary Information

Below is the link to the electronic supplementary material.

Supplementary Material 1

Supplementary Material 2

Supplementary Material 3

Supplementary Material 4

## Data Availability

Not applicable.

## Abbreviations

2-APB: 2-Aminoethoxydiphenyl borate
APL: Acute promyelocytic leukemia
As_2_O_3_: Arsenic trioxide
Cf: caffeine
CsA: Cyclosporin A
[Ca^2+^]_c_: Cytosolic concentration of the cation
DHR: Dihydrorhodamine 123
FBS: Fetal bovine serum
IP_3_R: Inositol 1,4,5-trisphosphate receptor
mitoO_2_^.−^: Mitochondrial superoxide
[Ca^2+^]_m_: Mitochondrial Ca^2+^ concentrations
MCU: Mitochondrial Ca^2+^ uniporter
MPT: Mitochondrial permeability transition
NOX 2: NADPH oxidase 2
PBS: Phosphate buffer saline
PMA: Phorbol-12-myristate-13-acetate
PML/RARα: Promyelocytic leukemia-retinoic acid receptor-α
ROS: Reactive oxygen species
Ry: Ryanodine
NaAsO_2_: Sodium arsenite
