# Supplementary material for: Distinct Ca2+ pools regulate NADPH oxidase 2 activation driving Ca2+-independent mitochondrial ROS formation and mitochondrial permeability transition in arsenic trioxide-treated NB4 cells
Source: Arch Toxicol. 2026 Apr 13;100(7):2995–3010. doi: 10.1007/s00204-026-04328-9 (PMC13309452; doi:10.1007/s00204-026-04328-9)
Supplement: Supplementary file 1 — Supplementary Material 1 [file 204_2026_4328_MOESM1_ESM.docx]

**Distinct Ca²⁺ Pools Regulate NADPH Oxidase 2 Activation Driving Ca²⁺-Independent Mitochondrial ROS Formation and Mitochondrial Permeability Transition in Arsenic Trioxide–Treated NB4 Cells**

Andrea Guidarelli*, Andrea Spina, Gloria Buffi, Mara Fiorani and Orazio Cantoni

*Department of Biomolecular Sciences, University of Urbino Carlo Bo, Urbino, Italy.*

***Corresponding author:** Prof. Andrea Guidarelli, Dipartimento di Scienze Biomolecolari, Sezione di Farmacologia e Igiene, Università degli Studi di Urbino, Via S. Chiara 27, 61029 Urbino (PU), Italy. Tel: +39-0722-303524; Fax: +39-0722-305470; e-mail: [andrea.guidarelli@uniurb.it](mailto:andrea.guidarelli@uniurb.it)

**Supplementary legend to the figuresù**

**Supplementary Figure S1. Characterization of the Ca^2+^ responses mediated by ATP or Cf in NB4 cells.**

Cells were pre-exposed for 20 min to Fluo 4-AM (A) or Rhod-2-AM (B) and then treated for a further 10 min with increasing concentrations of ATP, or Cf. Fluo 4- and Rhod 2-fluorescence was then quantified as detailed under Materials and Methods. The results represent the means ± SD calculated from at least 3 separate experiments. *P < 0.05, **P < 0.01, as compared to untreated cells (one-way ANOVA followed by Tukey test).

**Supplementary Figure S2. Time dependence of the apoptotic response induced by ATO**

NB4 cells were exposed for increasing time intervals to 1 µM ATO and then analyzed for apoptotic DNA fragmentation/condensation by the Hoechst assays. The results represent the means ± SD calculated from at least three distinct experiments. *P < 0.05, **P < 0.01, as compared to untreated cells (one-way ANOVA followed by Tukey test).

**Supplementary Figure S3. A 6 h exposure of NB4 cells to 2.5 µM NaAsO_2_ fails to promote phosphorylation of p47^phox^**

NB4 cells were treated for 6 h with 2.5 µM NaAsO_2_ or for 15 min with 0.162 µM PMA. After treatments, the cells were analyzed for phospho p47^phox^ expression. The relative band intensity of phospho p47^phox^ is depicted in the top bar chart. p47^phox^ was accounted as loading control. Results represent the means ± SD calculated from three separate experiments. **P < 0.01, compared with untreated cells (one-way ANOVA followed by Dunnett's test)
