# Supplementary figures and images for: Distinct Ca2+ pools regulate NADPH oxidase 2 activation driving Ca2+-independent mitochondrial ROS formation and mitochondrial permeability transition in arsenic trioxide-treated NB4 cells
Source: Arch Toxicol. 2026 Apr 13;100(7):2995–3010. doi: 10.1007/s00204-026-04328-9 (PMC13309452; doi:10.1007/s00204-026-04328-9)

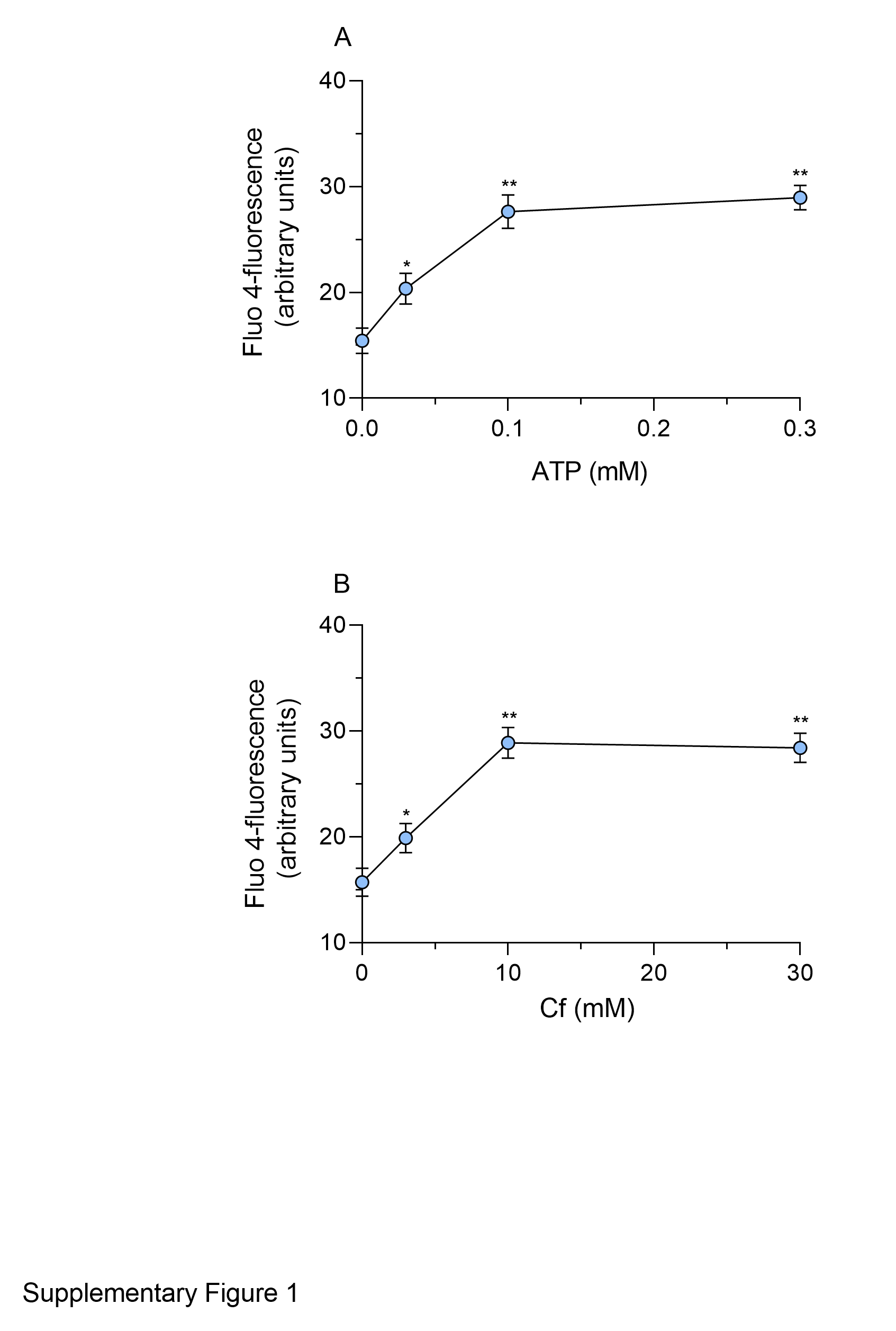

Supplement: Supplementary file 2 — Supplementary Material 2 [file 204_2026_4328_MOESM2_ESM.tif]

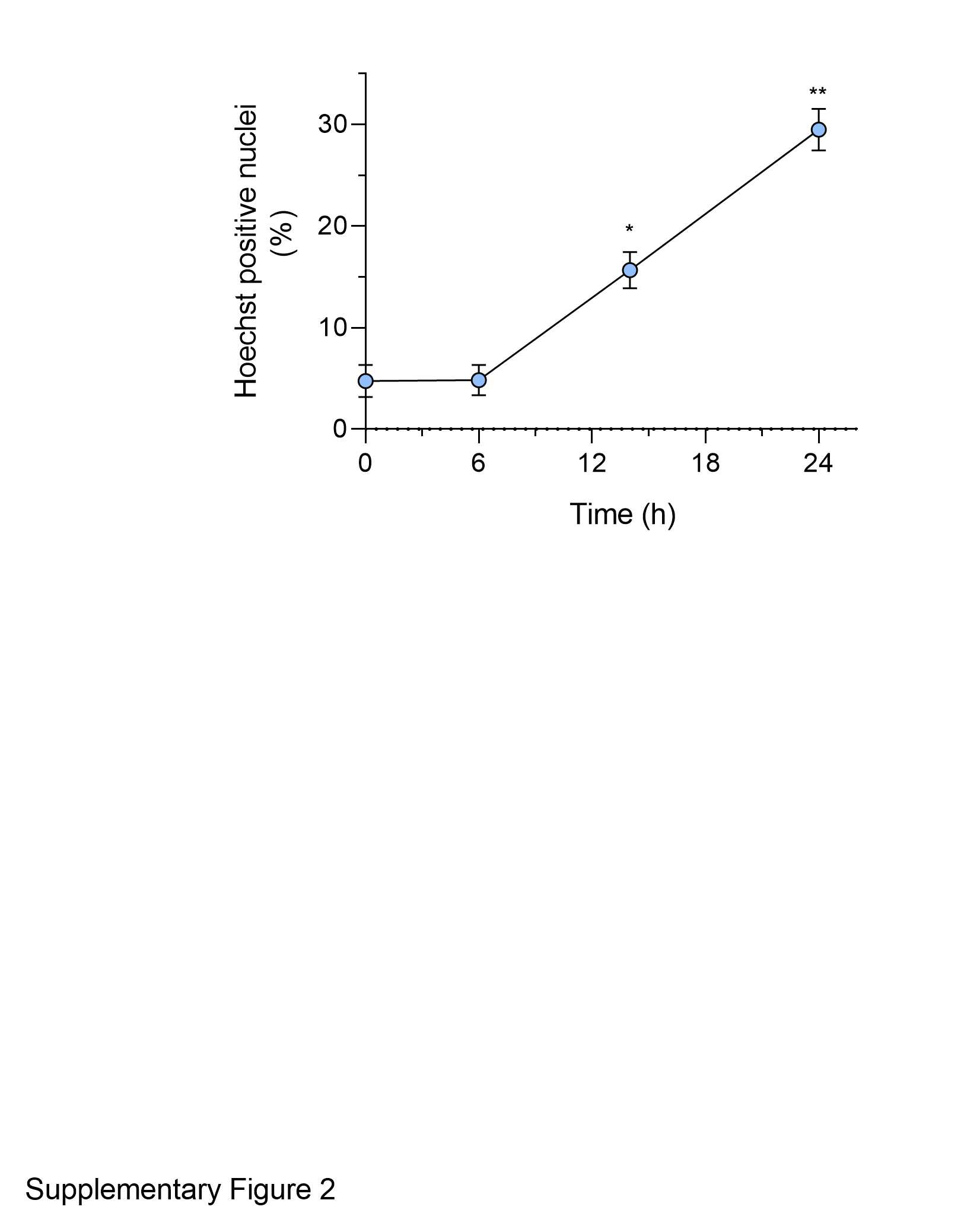

Supplement: Supplementary file 3 — Supplementary Material 3 [file 204_2026_4328_MOESM3_ESM.tif]

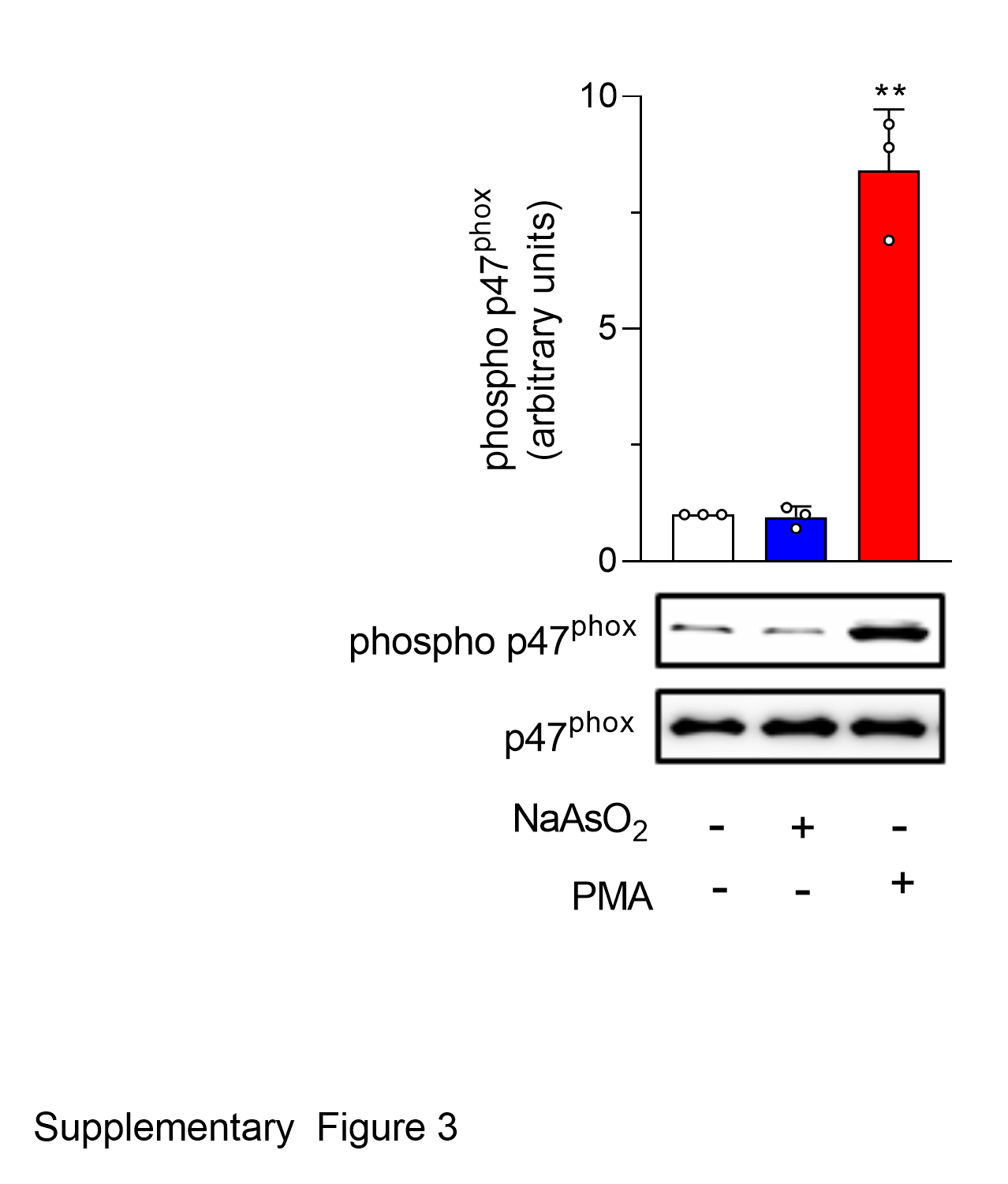

Supplement: Supplementary file 4 — Supplementary Material 4 [file 204_2026_4328_MOESM4_ESM.tif]
